# Case report: A first case of flaxseed-induced anaphylaxis in Korea

**DOI:** 10.1097/MD.0000000000008220

**Published:** 2017-12-08

**Authors:** Yewon Kang, So-Young Park, Soomin Noh, Jinyoung Kim, Bomi Seo, Oh Young Kwon, Hyouk-Soo Kwon, You Sook Cho, Hee-Bom Moon, Tae-Bum Kim

**Affiliations:** a Department of Internal Medicine; b Department of Allergy and Clinical Immunology, Asan Medical Center, University of Ulsan College of Medicine, Seoul, Republic of Korea.

**Keywords:** anaphylaxis, flax, food allergy, intradermal test

## Abstract

**Rationale:**

Anaphylaxis is a serious, generalized allergic reaction typically triggered by drugs, food, and bee venom, which can be life-threatening. Seeds are one of the major food allergens and can cause anaphylaxis as well as systemic hypersensitivity reactions. Flaxseed has been widely used crop for numerous purposes, such as in alternative medicine and as a dietary supplement, hypersensitivity to it has rarely been reported.

**Patient concerns:**

A 42-year-old female presenting with facial edema, dyspnea and urticaria after ingested half teaspoon of flaxseed flour 30 minutes previously.

**Diagnoses:**

A skin prick test for heated flaxseed flour extract showed negative responses, but intradermal test showed positivity which suggested an Immunoglobulin E-mediated reaction.

**Interventions:**

The patient was instructed to avoid future ingestion of flaxseed.

**Outcomes:**

The patient had no recurrence of symptoms at 1-year follow-up.

**Lessons:**

This is the first case of flaxseed-induced anaphylaxis in Korea, confirmed by an intradermal skin test.

## 1 Introduction

Flax (*Linum usitatissimum*), also known as linseed, is an annual plant of Central Asian and Arabian origin, which is widely grown all over the world and is commercially important. Flax fiber has been used in the textile industry and the oil from its seed has been used as an ingredient for printing ink, paints, and varnishes since ancient times. Flaxseed is also used in baking products and as feed for animals and poultry. Flaxseed oil has been used in various folk remedies for wound healing, skin moisturizing, and constipation relief.^[1,2]^ Recently, flaxseed has been introduced as a so-called super food and its use in alternative medicine and as a dietary supplement has been increasing in Korea.^[3]^

Since 1930, when Black^[4]^ reported the first case of flaxseed anaphylaxis, few hypersensitivity cases have been reported. Most of these reported cases involved anaphylaxis, with occupational asthma^[5]^ and contact dermatitis also reported.^[6]^

Here, we report the first diagnosed case of anaphylaxis caused by the ingestion of flaxseed flour in Korea, confirmed by an intradermal skin test, as well as a literature review. Patient gave informed consent for the case presentation in accordance with the Declaration of Helsinki.

## 2 Case presentation

A 42-year-old female presenting with facial swelling, shortness of breath, and whole body urticaria was referred to our emergency department. She had eaten a green apple, a tomato, and half a teaspoon of flaxseed flour 30 minutes previously. She had not taken any medication and her initial blood pressure was 144/86 mm Hg. Her respiratory rate was 24/min, her pulse rate was 120/min, and she had an oxygen saturation of 100%. We administrated epinephrine and chlorpheniramine, as her dyspnea, flushing, and edema were ongoing and she appeared acutely unwell, as we considered her presentation was consistent with an anaphylactic reaction. Two hours later, her dyspnea and skin symptoms improved. Four weeks later, she was referred to the outpatient clinic for further tests to culprit of the anaphylaxis.

Her medical history included hypertension, allergic rhinitis, and a history of hospitalization with anaphylaxis caused by cefaclor 6 months previously, but this most recent event was not related to cefaclor. Four weeks later, the results of serum total immunoglobulin E (IgE) and specific immunoglobulin E tests for green apple and tomato were within the normal range. Skin prick tests were conducted on 30 food allergens and 40 inhalant allergens. Histamine and saline were tested as positive and negative controls, respectively. All 30 food allergens including apple and tomatoes were negative. Among the 40 inhalant allergens, test results were positive for *D. farinae, D. pteronyssinus*, *Tyrophagus,* and *Trichophyton*, with wheal reactions of 4 mm × 4 mm, 5 mm × 5 mm, 5 mm × 5 mm, and 5 mm × 5 mm, respectively. Therefore, we decided to test the skin reaction, suspecting that flaxseed flour as the most probable cause of anaphylaxis.

**Figure 1:**
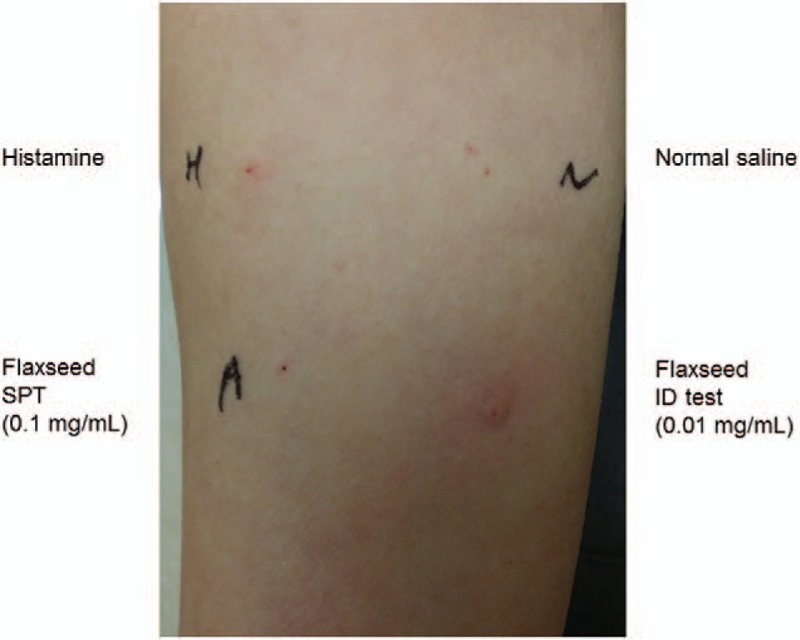
The positive result of intradermal skin test. The prick-to-prick test is negative but the intradermal test showed a positive response to flaxseed. ID = intradermal, SPT = skin prick test.

First, a skin prick test was performed using flaxseed flour extract. Flaxseed flour (1 mg) was mixed with 10 mL of sterile normal saline and centrifuged to collect the supernatant. A skin prick test was performed with this solution, and showed a 2 mm × 2 mm wheal and 2 mm × 2 mm flare. However, the wheal and the flare of the positive control (histamine) measured 3 mm × 3 mm and 4 mm × 4 mm, respectively, while the dilution of flaxseed solution was confirmed to be negative. Therefore, we conducted an intradermal skin test with the flaxseed extract solution, diluted at 1:10 from the previously prepared solution. Fifteen minutes after administering the intradermal skin test, a positive reaction was confirmed with a wheal of 5 mm × 5 mm and a flare of 20 mm × 20 mm (Fig. 1 and Table 1). To exclude the possibility of skin irritation and avoid a false positive test result, intradermal tests were administered to healthy control and the result was negative.

**Table 1:**
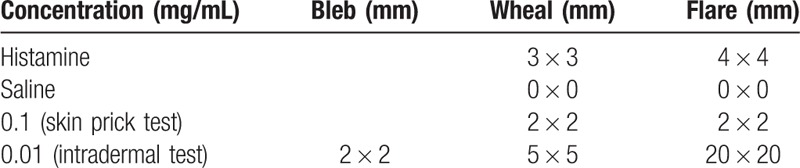
The result of skin test to flaxseed extract.

As a result of these tests, the patient was diagnosed with flaxseed hypersensitivity and was instructed to avoid future consumption of flaxseed.

## 3 Discussion

Anaphylaxis is an acute, potentially fatal, systemic hypersensitivity reaction caused by type 1 hypersensitivity. The common causes of anaphylactic reactions are food, drugs, and bee venom, of which anaphylactic reactions due to food allergens are rapidly increasing.^[7]^

In cases of anaphylaxis caused by nuts and seeds, peanuts and sesame seeds are the most common causes. Other cases of perilla, poppy, sunflower, and mustard seed anaphylaxis have also been reported.^[8]^ Flaxseed has rarely been reported as a sensitizer, and there have been few reports since 1930. Clinical cases are related to seeds,^[9,10]^ multigrain bread,^[11,12]^ or to flaxseed oil^[13]^ used as a laxative. There have been reports of occupational asthma and contact dermatitis as well as anaphylaxis cases.

Flaxseed and flaxseed derivative are rich sources of α-linolenic acid, a precursor of omega-3 fatty acids, and have been associated with an anti-inflammatory, anti-oxidant effect, and a cardioprotective action.^[14]^ The lignan of flaxseed, a type of plant estrogen, possesses antioxidant properties, and is reported to reduce the risk of breast and prostate cancer.^[1,15]^ For these reasons, flaxseed has recently become one of the most popular super foods.

Allergenicity to flaxseed has been studied in several cases. Alonso et al^[13]^ identified 20, 22, 30, 35, and 38 kilodalton (kDa) IgE binding proteins via sodium dodecyl sulfate polyacrylamide gel electrophoresis (SDS-PAGE) with IgE immunoblotting. Leon et al^[16]^ described a 56 kDa dimeric protein and reported that 35 kDa malate dehydrogenase-1 in flaxseed suggested a major allergen. Similar to the 56 kDa fraction, the 53 kDa band protein was detected in a study by Alvarez-Perea et al.^[9]^

Fremont et al^[17]^ also found IgE binding 25 and 38 kDa protein bands. In addition, they studied the cross-reactivity of flaxseed with other seeds. In a prospective study of 1317 patients visiting an allergy clinic, a prick-to-prick test showed that 77 (5.8%) patients were sensitized to flaxseed and 73 had a history of atopic disease. Two of the 77 responders showed a hypersensitive reaction, with 1 patient presenting with anaphylaxis and 1 patient presenting with angioedema.^[17]^ Serologic studies from flaxseed-sensitive patients showed various degrees of cross-reactivity between flaxseed and lupine, peanut, soybean, rapeseed, rape pollen, and wheat, but the clinical relevance was not clarified.^[18]^

Our patient in this case had a history of allergic rhinitis and cefaclor anaphylaxis. A prick to prick test for heated flaxseed flour extract showed negative responses, but positivity in the intradermal test suggested an IgE-mediated hypersensitivity reaction. The oral provocation test was not performed considering the risk of anaphylaxis.

Flaxseed has been an important crop since ancient times, and a variety of its benefits have been studied in recent years and its use as food, medicine, and as an alternative medicine is increasing. Hence, we believe that flaxseed hypersensitivity reactions, and cross-reactivity with other seeds could be increased, and further studies are needed to explore this issue fully.
